# Evaluation of the coupling coordination effect of Hefei Rail transit station area based on supply and demand matching

**DOI:** 10.1371/journal.pone.0322856

**Published:** 2025-05-15

**Authors:** Wangyang Gui, Mengnan Cheng, Tong Zhang, Yifei Guo, Bin Xu

**Affiliations:** 1 School of Architecture and Planning, Anhui Jianzhu University, Hefei, Anhui, China; 2 China Architectural Design and Research Institute Limited Local Design Research Center, Beijing, China; The University of Tokyo, JAPAN

## Abstract

The coupling of supply and demand in the rail transit station area is an important driving force for ensuring the balanced development between the traffic functions and the place performance of the rail transit station area. This study evaluates the development status of these two aspects in each rail transit station area explores the possibility of combining the TOD (Transit-Oriented Development) model with the concept of the 15-minute city, and focus on the importance of TOD planning and its optimization strategies, this paper takes 44 rail transit station areas within the Second Ring Road of Hefei City as examples. Through multi-source data, a “supply-demand” coupling coordination degree model is constructed to evaluate the matching status between the needs of “people” and the supply of facilities and resources in the rail transit station area, and analyze the influence mechanism of the allocation of public service resources in different communities on the rail transit station area, thus constructing a comprehensive analysis framework and practical guidelines for modern urban planning. For the station areas with poor coupling coordination of supply and demand, references are provided for urban planning from four dimensions: the construction of the planning reference framework, the scientific nature of the early-stage site selection, the dynamic adjustment of the mid-term monitoring, and the refinement of the later-stage governance, which is of great significance for promoting the comprehensive development and sustainable development of the areas surrounding the rail transit stations.

## Introduction

Faced with the “big city disease” of traffic congestion, environmental pollution, and resource scarcity, as well as the “urban community life disease” of separation of work and residence, insufficient supporting facilities, and lack of sense of belonging, China’s previous rail transit station area based on station development and traditional residential area planning can no longer meet people’s increasingly diverse living needs [1–3]. In the process of modern urban development, the TOD (Transit-Oriented Development) concept is highly compatible with the widely implemented 15-minute city concept. At the same time, the TOD model centers around public transportation stations, creating urban space development areas within a 5–15 minute walking radius. The core development area of this radius often overlaps with the scope of the 15-minute city. Based on this context, the coordinated development of transit-oriented station areas and the 15-minute living circle has become a key driving force for reshaping urban space in the new era [4–5]. Establishing an effective research methodology to evaluate the current development status of rail transit station areas, exploring the Possibility of Integrating the TOD Model with the 15-Minute City Concept, scientifically fostering synergy between rail construction and the allocation of community public service resources, enhancing their respective efficiencies, and maximizing their combined effectiveness has emerged as a pressing research agenda. This agenda aims to accommodate the diverse needs of citizens and ensure the sustainable development of rail transit.

## Literature review

Firstly, the interaction between rail transit station areas and urban spaces involves both “interactive feedback” and “source-flow” relationships. Rail transit influences urban land value, utilization structure, and intensity, while land use patterns are crucial factors in increasing rail transit ridership. Schaeffer and Sclar highlighted the role of urban transportation systems in the evolution of urban spatial morphology [6]. Moon through research on subway stations in the San Francisco Bay Area and Washington, D.C., demonstrated that suburban stations significantly contribute to the development and construction of adjacent commercial land [7]. The Transportation Cooperative Research Program (TCRP) in 1995, in its study of rail transit stations in the United States and Canada, revealed that a 10% increase in population density leads to a 6% increase in traffic volume [8]. Cervero proposed that high-density land use patterns can enhance public transport ridership and are key to stimulating urban rail ridership [9]. Cervero found that increased land use density within a 1,600-meter radius of Bay Area urban rail stations can lead to higher passenger volumes [10]. Bowes et al. showed that rail transit stations can increase the value of surrounding land by reducing commuting costs and attracting retail activities [11]. Lu Jiwei et al. suggested that rail transit can effectively promote regional intensification, functional efficiency, and the development of underground space [12]. Cervero and Kang studied the impact of Seoul’s public transportation system on urban land prices in Korea, demonstrating that improvements in urban transportation conditions increase urban land price premiums [13]. Cui Xu et al. through an analysis of Chengdu Guanxian Express Railway stations, concluded that the expansion of residential land around rail transit is most significant, while commercial land tends to concentrate near stations [14].

Secondly, research on rail transit station areas primarily adopts an urban perspective, focusing on how rail transit can better serve urban development, planning, and layout. Yuan M integrated considerations of urban design and space utilization, establishing a spatial utilization analysis method that emphasizes the fundamental context, user characteristics, and spatial utilization distribution of rail transit station areas [15]. Zhang Y examined the variations in job-housing spatial patterns across stations in different locations, combining these insights with human needs for living and working, to further optimize the construction of rail transit station areas [16]. Shen Hongtian investigated renewal strategies from the perspective of urban catalysts, analyzing the characteristics, existing challenges, renewal status, and goals of each type of station area, and proposing corresponding implementation strategies for renewal [17]. Tu et al. employed the least squares method and spatial lag model to study the correlation between vitality and the built environment of subway stations in Shenzhen, Singapore, and London [18]. Xiao et al. utilized a two-layer graph convolutional neural network model to predict the contribution of the built environment in subway station areas to the vitality of those areas [19]. Based on this, they explored the nonlinear relationships and synergistic effects between the built environment and station vitality, using Shenzhen’s rail transit stations as empirical cases.

Furthermore, the rail transit station area represents an urban space characterized by multiple dimensions, including geographical, social, and individual perceptions. Internationally, cities such as Sacramento in the United States, Florida, and Edmonton in Canada have proposed diverse evaluation indicators for urban rail transit construction within their urban planning guidelines [20–22]. Caset, F. utilized node-place modeling to conduct a quantitative analysis and empirical assessment of the transportation and land use characteristics of all RER stations, concluding that accessibility can effectively identify differentiated development among RER stations [23]. Qin Yuchen et al. employed the fuzzy comprehensive evaluation method to create a membership degree matrix for assessing the spatial performance of the connections between stations and commercial complexes [24]. Su Ying examined the integration of rail transit complexes with slow traffic, public transportation, and private car traffic through participatory observation and semi-structured interviews [25]. Overall, current comprehensive evaluation research on rail transit station areas primarily emphasizes aspects such as traffic accessibility, spatial use performance, agglomeration effects, and land performance [26–28]. Evaluation methods include Bayesian networks, multi-source data support, and multi-semantic feature analysis, among others [29–31]. However, there remains a lack of quantitative analysis regarding the configuration of public service facilities.

### Research Subject

**Fig 1:**
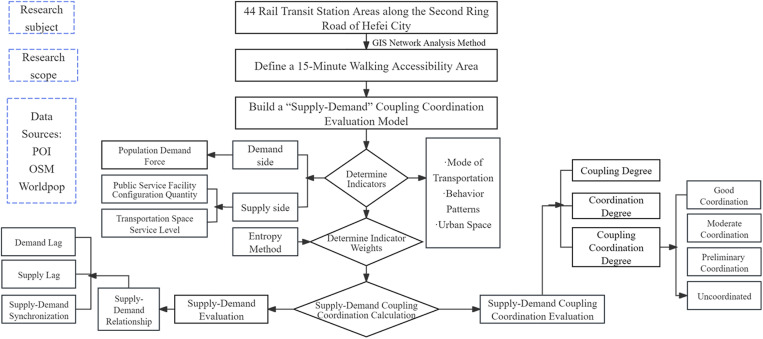
Research Framework Diagram.

Through the above research on the development and related evaluation methods of domestic and foreign rail transit station areas, it is found that, on the one hand, there is insufficient research on the supply - demand balance of rail transit station areas. Here, supply refers to the service level provided by public service facilities and transportation space within transit - oriented station areas, while demand is represented by the dynamic needs of the resident population. Specifically, it is the urgent demand of residents for regional services, focusing on population vitality and economic activity. The lack of such research can easily lead to mismatched land division and subsequent unreasonable resource allocation in rail transit station areas. On the other hand, the evaluation of rail transit station areas needs to be refined. Most evaluation models focus on the urban perspective and lack the perspective of human demand. Further quantitative evaluation and analysis are needed to understand the coupling and coordinated development of rail transit station areas at the supply and demand level in different development stages, as well as the spatial distribution characteristics and underlying causes of different types of rail transit station areas. This study takes the rail transit station area in Hefei City as the research object, constructs a supply-demand evaluation system for the rail transit station area, evaluates the coupling coordination effect and development characteristics of each rail transit station area, explores the technical methods, construction paths, and implementation mechanisms of rail transit station area planning, provides the “optimal solution” for convenient travel and comfortable life of residents, and provides samples for solving the “big city disease” in China (Fig 1).

### Research scope and data sources

#### Research scope.

**Fig 2:**
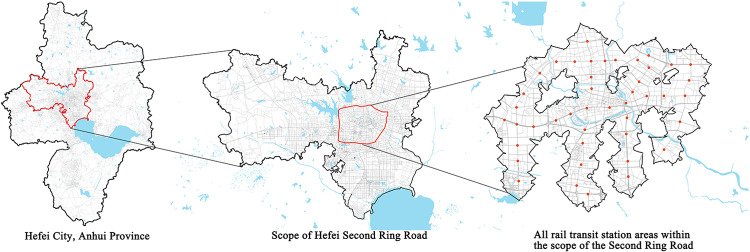
Scope Map of Research Object.

Hefei, as a new first-tier city at the intersection of Anhui’s three major national strategies of integrated development in the Yangtze River Delta, Yangtze River Economic Belt, and high-quality development in the central region, has developed rapidly. In the past 10 years, it has planned 11 rail transit lines and has already operated 5 of them, with a total length of 210 kilometers and 161 stations along the entire line. It covers a wide range of types and has a good sample effect on cities under construction of rail transit (Fig 2).However, despite its progress in rail transit development, Hefei’s 15-minute city initiative is still in its early stages, unable to fully meet residents’ demands for a high-quality urban life. As such, accelerating the development of the TOD (Transit-Oriented Development) model has become an urgent priority. Hefei represents a typical case of urban development in China, and the challenges it faces in TOD planning are not unique. These issues are shared by many emerging first-tier cities in China and will also be faced by other regions striving to transition into new first-tier cities in the future.

This article selects all rail transit stations within the Second Ring Road in Hefei City as the research object, as a typical case for empirical analysis in the study of urban rail transit station areas. Firstly, the urban location and spatial characteristics are prominent. The Second Ring Road, as an important supporting part of the urban spatial structure system, covers the spatial construction of the old and new urban areas, as well as the core areas of the four districts of Shushan, Yaohai, Luyang, and Baohe; The second reason is that the functional mixing of surrounding land is relatively high, and the construction time of the stations is relatively early, which has already had a certain driving effect on the renewal and renovation of the surrounding areas. Moreover, most of the stations are ordinary mixed-type stations, rather than urban areas with relatively single functions. This research has general applicability for future studies and provides greater reference value for the study of station areas in modern cities, both in China and globally. In the study, the center of the rail transit station was taken as the measurement point, and the walking speed of pedestrians in their daily lives was calculated as 1.2m/s ~ 1.4m/s. The service area analysis tool of the GIS network analysis module was used to simulate the 15-minute walking range of the station to delineate the research scope of this article.

### Data sources and processing

This study includes multi-source data such as population, road network, and public service facilities. The main data sources and processing methods involved are as follows: POI data: POI data is obtained from all public service facilities within the research scope through the API interface of Gaode Map combined with Python programming programs. According to the 2018 version of the “Urban Residential Area Planning and Design Standards” on the setting of supporting facilities in the 15-minute city residential area, the obtained data is filtered and the corresponding relationship between POI data subcategories and supporting facility classifications is established. Road network data: Obtain open-source map data of traffic facilities and urban building environment in Hefei city through the OpenStreetMap website, filter and remove all roads that prohibit pedestrian traffic, further improve the intersection data through merging and interrupting road network data, and finally use topology rules to check and verify the roads in the study area, reducing the occurrence of errors WorldPop Population Grid: Using the WorldPop website to obtain high-resolution image information grid data of the population of Hefei City with a resolution of 100 m by 100 m, convert it from the geographic coordinate system to the UTM (Universal Transform Mercator) projection coordinate system, and perform projection calculations based on the distribution zones of different new areas.

### Research methods - index system construction

#### Indicator screening.

**Fig 3:**
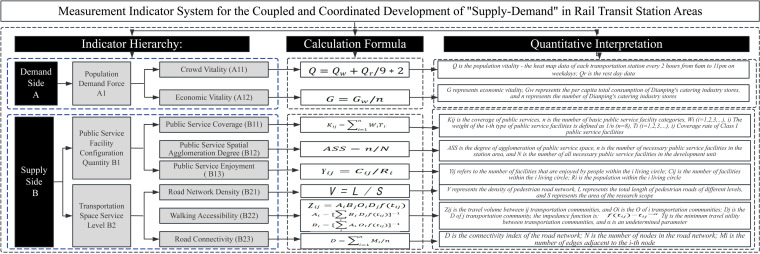
Measurement Index System.

Based on combining relevant national regulations and standards,existing evaluation indicators for supply-demand balance systems in domestic and foreign 15-minute city, and existing scientific research achievements, this study selects influential factors with high frequency of use and representativeness to establish a coupling coordination index system for public service facilities supply and demand, and processes and visualizes these data. The study proposes 8 evaluation criteria based on the two dimensions of supply and demand. The demand represents the demand capacity of the permanent population, specifically the urgency of residents’ need for regional services. It focuses on the dynamic demands of the population, which can be summarized as population vitality and economic vitality. The supply represents the service level provided by the transit-oriented station areas, specifically whether the regional services meet the demands of the population in that area. This is mainly assessed in terms of public service facility provision and transportation space services. It is calculated based on the public service coverage,public service spatial agglomeration degree, public service enjoyment,road network density,walking accessibility and road connectivity (Fig 3).

#### Determining weights.

Due to the varying degrees of impact of various indicators on the coordination of supply and demand coupling of public service facilities, this study uses the entropy method for weight analysis to quantitatively evaluate the spatial adaptability of rail transit stations. The specific calculation steps are:

Calculate the entropy value of the second indicator:

$$e_{j}=-\frac{1}{lnm}\sum_{i=1}^{m}P_{ij}\ln\left(P_{ij}\right)$$ (1)

In the formula, $P_{i}j$ is the characteristic proportion of the j indicator in the i evaluation object,


$$P_{ij}=-\frac{X_{ij}}{\sum_{i=1}^{m}X_{ij}}$$ (2)

Calculate the weight of the j indicator:

$$W_{j}=\frac{1-e_{j}}{\sum_{i=1}^{n}\left(1-e_{j}\right)}$$ (3)

The specific index weight results of all levels are as follows Table 1.

**Table 1:** Weight value of the “supply-demand” coupling coordination evaluation index.

Primary indicators	Secondary indicators	Indicator weight	Third level indicators	Indicator weight
Demand Side A	Population Demand Force A1	1	Crowd Vitality (A11)	0.3555
			Economic Vitality (A12)	0.6445
Supply Side B	Public Service Facility Configuration Quantity B1	0.6646	Public Service Coverage (B11)	0.0774
			Public Service Spatial Agglomeration Degree (B12)	0.1162
			Public Service Enjoyment (B13)	0.4711
	Transportation Space Service Level B2	0.3354	Road Network Density (B21)	0.1639
			Walking Accessibility (B22)	0.0782
			Road Connectivity (B23)	0.0933

### Building a “supply-demand” coupled coordination calculation model

Based on a fuzzy comprehensive evaluation, the comprehensive scoring results R1 and R2 are defined to represent the specific performance of the rail transit station area in the “supply-demand” two indicator layers. Taking the R1 indicator layer of crowd demand as an example, the following formula is used to illustrate:


$$R_{1}=S_{A11}T_{1}+S_{A12}T_{2}$$ (4)

Among them, $S_{A11}andS_{A12}$ respectively represent the score data obtained by using the min-max standardization method for the population vitalityA11 and economic vitalityA12 under the indicator layer, $T_{1}andT_{2}$ represent the corresponding indicator weights. Since the two primary indicator layers have the same importance, the sum of the lowest-level indicators under their indicator layers is 1, as shown in the following equation:


$$T_{1}+T_{2}=1$$ (5)

### Evaluation of supply demand coupling coordination in rail transit station area

The calculation steps for the coupling coordination degree of the rail transit station area are as follows:1.Calculate the Coupling Coordination Development Index (CDI), which consists of the Development Index (DI) and the Coupling Coordination Index (CI). The formula for calculating the Coupling Coordination Index (CI) is:


$$CI=2\cdot {\left[\frac{f\left(x\right)\cdot g\left(y\right)}{{\left(f\left(x\right)+g\left(y\right)\right)}^{2}}\right]}^{1/2}$$ (6)

In the formula, f(x) and g(y) is the comprehensive score corresponding to the two index layers of “supply-demand” respectively Take as f(x) an example:


$$f\left(x\right)=\sum_{i=1}^{k}w_{i}x_{i}$$ (7)

Where w is the weight of the i three-level index within the two index layers; k is the sum of the number of indicators of the three-level index layer;x is the result of the i-index standardization within the three-level index layer. The development index (DI) is calculated as follows:


DI=αf(x)+βg(y) (8)

Where α and β are the corresponding weights. Since the three index layers have the same importance, they are set to 1/ 2 in this study. Therefore, the coupled coordinated development index (CDI) can be found in the substitution formula:


$$CDI=\sqrt{CI\cdot DI}$$ (9)

2. Determine the evaluation criteria for coupling coordination. To more accurately characterize the performance characteristics of supply equilibrium within each rail transit station area, based on the spatial calculation results of the coupled coordinated development index, and referring to the existing research results on the classification method of the coupling coordination index level of the “supply-demand” system, the “four-part method” is selected to establish a classification system and evaluation criteria for the coupling coordinated development of the supply-demand system [32–33]. The specific situation is as follows Table 2:

**Table 2:** Classification Criteria for the Degree of Coupling and Coordination.

Degree of Coupling and Coordination (CDI)	Classification	Relationship between f(x) and g(y)	Basic Type
0.000-0.250	Uncoordinated	f(x) > g(y) f(x) = g(y) f(x) < g(y)	Supply Lag type Supply-Demand Synchronization type Demand Lag type
0.251-0.500	Preliminary Coordination	f(x) > g(y) f(x) = g(y) f(x) < g(y)	Supply Lag type Supply-Demand Synchronization type Demand Lag type
0.501-0.750	Moderate Coordination	f(x) > g(y) f(x) = g(y) f(x) < g(y)	Supply Lag type Supply-Demand Synchronization type Demand Lag type
0.751-1.000	Good Coordination	f(x) > g(y) f(x) = g(y) f(x) < g(y)	Supply Lag type Supply-Demand Synchronization type Demand Lag type

Calculation of “supply-demand” coupling coordination within the rail transit station area of Hefei Second Ring Road

Score the situation of the supply side and demand side

(1) Demand side score situation

**Fig 4:**
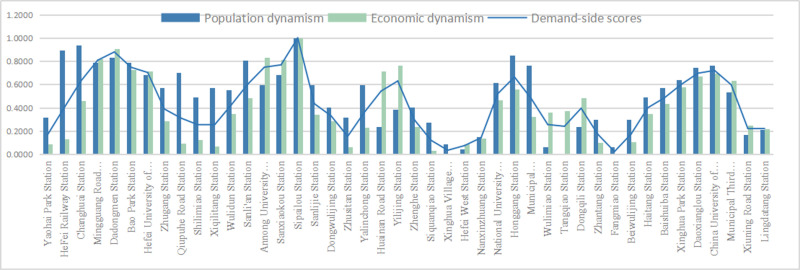
The demand side score.

**Fig 5:**
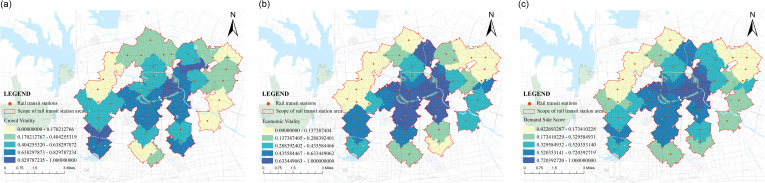
Demand side score classification distribution chart.

The demand side represents the demand of the population, that is, the degree to which residents urgently need regional services. It focuses on the dynamic needs of the population. Based on the characteristics of population size, economic level, etc., this study selects two activity indicators, including crowd vitality and economic vitality. In the calculation of the built space of various rail transit stations within the second ring road in Hefei, it was found that the overall score on the demand side was higher than the score on the supply side (Fig 4); The highest score is 1.00 for Sipailou Station. As the largest urban core commercial district in Hefei, it has been developed for a long time and has a mature surrounding environment, which gives it good economic and population vitality. The minimum score is 0.02 for Fangmiao Station, which is mainly composed of industrial and residential land within the station area. The development level of surrounding land is low, and emphasis is placed on industrial clustering, transportation support, and basic living needs support. The crowd aggregation effect is low, and the corresponding economic vitality is also reduced. The score difference between the two is as high as 0.98, further indicating that there is a significant gap between the built environments of each station and the problem of uneven development. Based on the demand side scores of each rail transit station area, the natural breakpoint grading method in Arc GIS software was used to divide them into 5 levels, and their score values were divided at equal intervals (Fig 5). It was found that the demand side score in space showed a phenomenon of being centered around the Sanxiaokou Sipailou commercial district, with lower scores as the area is closer to the urban edge. The large natural boundaries on the northwest side, which are affected by a large amount of water area in the natural environment and cannot be crossed, limit the possibility of urban development in that direction, resulting in low scores; On the northeast side, due to the scarcity of surrounding land types, some land types are mainly industrial land, with uneven distribution of land and unreasonable resource allocation, resulting in the inability to meet the basic living needs of the population.

(2) Score situation on the supply side

**Fig 6:**
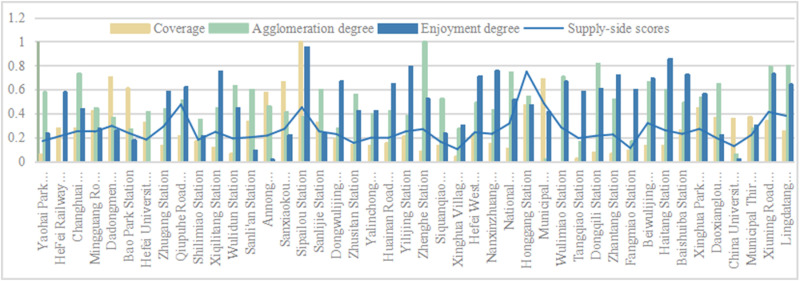
The supply side score-Public Service Facility Configuration Quantity Bl.

**Fig 7:**
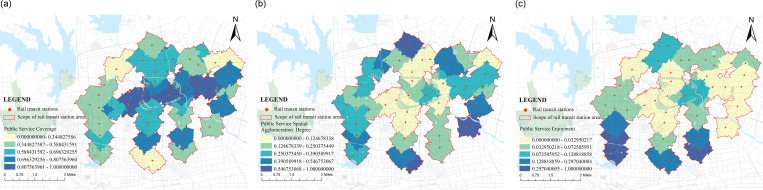
Supply side distribution chart of public service facility configuration quantity B1.

**Fig 8:**
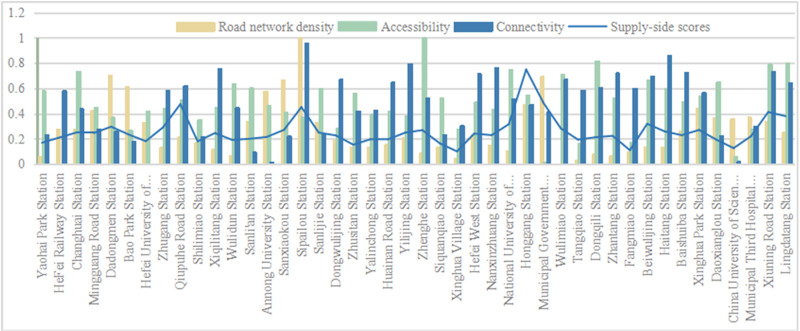
The supply side score-Transportation Space Service Level B2.

**Fig 9:**
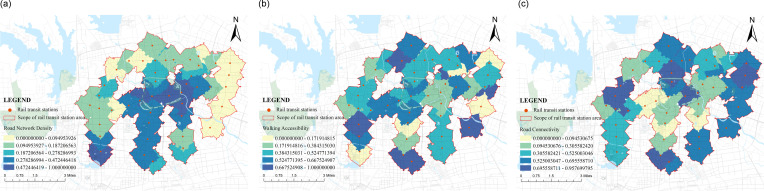
Supply side distribution chart of transportation space service level B2.

The supply side represents the service level of the rail transit station area, that is, whether the regional services meet the needs of the population in that area. The main considerations are the allocation of public service facility configuration quantity and transportation space service level. Taking into account the quantity, scale, and layout, the public service coverage,public service spatial agglomeration degree and public service enjoyment are calculated. The service level of transportation space is evaluated comprehensively from the aspects of efficiency, scope, and quality, including road network density,walking accessibility and road connectivity. In the calculation of the supply side of the research object (Fig 6 and 8), it was found that the highest score was 0.75 for Honggang Station; The lowest score was 0.10 for Xinghua Village. Firstly, in terms of road planning, Honggang Station is a chessboard-style road system with high intersection density and strong permeability, which improves passing efficiency. There is an effective connection between pedestrians and destinations, which is one of the important reasons for its high supply level; Secondly, in terms of public facility configuration, the development intensity of the Honggang Station rail transit station area is high, with complete and evenly distributed basic public service facilities, and high per capita enjoyment, providing strong support for the high supply level of the station area; The Xinghua Village Station is affected by the natural environment, with the rail transit station mainly consisting of enclaves and an incomplete road system, which limits further land development and creates an opposite environmental state to Honggang Station. The difference in score between the two is 0.65, which is different from the demand side’s 0.98. This value indicates that the overall supply level of the station area is not high, but the development is relatively balanced. Using the natural breakpoint grading method in Arc GIS software, the supply side scores were divided at equal intervals (Fig 7 and 9), and it was found that high zoning was mainly distributed in the central and southern parts of urban functional clusters, showing linear development; The railway station is located in the northeast, and industrial development is the main focus along the railway line. The population attraction of this area is not high, the development intensity is small, and the supply level is low. Along the central and southern regions, there are many universities distributed, which occupy a large area. On-campus development mainly focuses on teaching and research, with the main goal of meeting the basic living needs of students. There is a lack of sharing with the surrounding population, resulting in a low overall supply-side score.

### Score of the development degree of “supply-demand” coupling coordination

**Fig 10:**
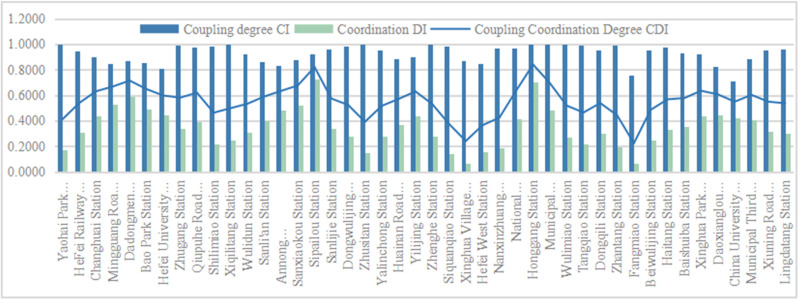
Score of coupling coordination degree.

**Fig 11:**
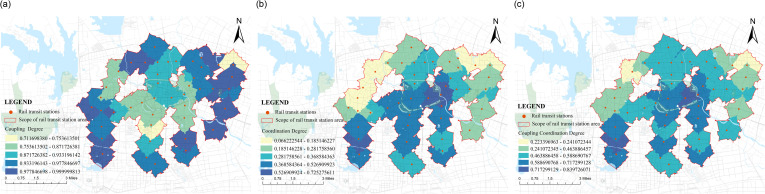
Distribution chart of coupling coordination degree.

The coupling and coordinated development index (CDI) is composed of the CI coupling degree and DI coordination degree. Based on the scores of the demand side and supply side mentioned above, this study calculated the coupling and coordination degree scores of the Hefei Second Ring Line rail transit station area (Fig 10). It was found that the lowest CI score for each rail transit station area was 0.71 for the East District Station of the University of Science and Technology of China, and the highest score was Yaohai Park Station, Xiqilitang Station, Zhusitan Station, and Municipal Government Center Station, with a score of 1.00, indicating that the interaction between the supply and demand systems of each rail transit station area is strong and the degree of influence is high. The lowest DI score is 0.07 for Fangmiao Station, and the highest score is 0.73 for Sipailou Station, indicating a greater degree of benign coupling. The highest CDI score is 0.84 for Honggang Station, and the lowest is 0.22 for Fangmiao Station. From the above analysis, it can be seen that Honggang Station is in an excellent state in road planning and public service facility configuration. In addition, it’s good population vitality and economic vitality make it a high-scoring state on both the supply and demand sides, and the development of the two systems is coordinated. The Fangmiao Station is affected by the nature of the land use, resulting in low demand from the population. The construction of related supporting facilities does not match the demand of the population, leading to a low degree of coupled and coordinated development. After using the natural breakpoint grading method in Arc GIS software to divide the coupling coordination score into equal intervals (Fig 10), it was found that CI and DI showed an opposite state, with CI gradually decreasing from the edge to the center (Fig 11a) and DI showing an increasing trend from the edge to the center (Fig 11b). From the overall CDI score situation (Fig 11c), with Sipailou Station and Honggang Station as the two main cores, the core edge gradually decreasing state was observed. Based on the scores of the supply side and demand side in the previous text, it can be seen that the spatial distribution of the score of coupled coordinated development roughly overlaps with the score of the supply side, which also indirectly illustrates the significant difference in the level of supply and demand in the rail transit station area.

## Results

### Grading characteristics of coupling and coordinated development degree

**Fig 12:**
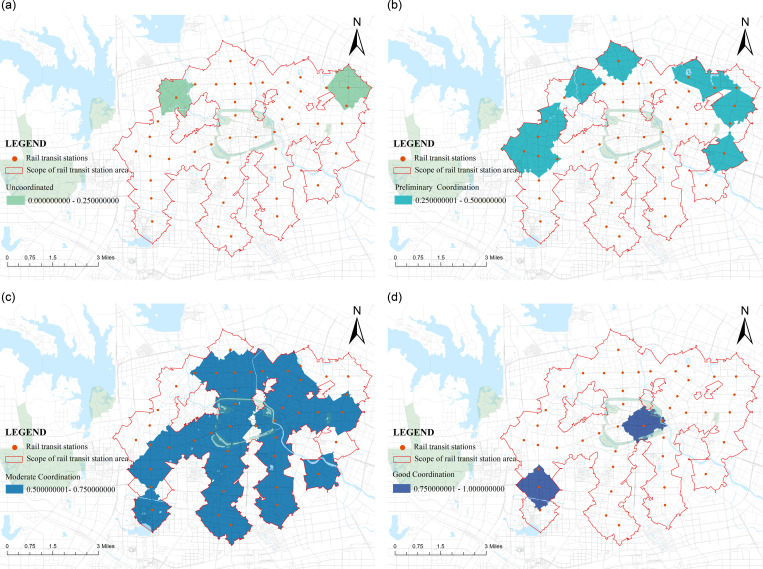
Distribution map of station coupling and coordination status.

Based on drawing on the research results of other scholars, this study, based on the CDI scores of each rail transit station area within the second ring road in Hefei City calculated in the previous text, divides each rail transit station area into four levels: uncoordinated, preliminary coordination, moderate coordination and good coordination. Among them, two levels belong to the high-quality coordinated development category, accounting for 5% of the total, namely Sipailou Station and Honggang Station. Among them, Sipailou Station scored 0.82 and Honggang Station scored 0.84. According to the spatial distribution of this type of station area (Fig 12a), although both are good coordination, Sipailou Station, as a mature commercial circle in Hefei City, has a population vitality and economic vitality. Their performance is extremely active, and they are more inclined towards high-quality development on the demand side. As a rail transit station area with high land development intensity in the surrounding area, Honggang Station has a complete road transportation system, diverse public facility configurations, and a reasonable layout to meet the various living needs of the population in the area, making it more inclined towards high-quality development on the supply side.

There are a total of 30 stations belonging to the category of moderate coordination, accounting for 68% of the total. According to their distribution (Fig 12b), it can be seen that the stations of well-coordinated development are mainly concentrated between Line 1 and Line 5 of the rail transit, forming a fragmented development state with Sanxiaokou Station, Dadongmen Station, and National University of Defense Technology Station as the core. However, due to differences in planning strategies, development models, etc., the “Sanxiaokou Core Area” is mainly focused on commercial development, which stimulates potential consumption among the population and belongs to the modern area. The core area of Dadongmen is located in the old urban area, with a mature built environment and more concentrated urban functional areas. The road traffic is mainly narrow and dense, which increases traffic efficiency, reduces travel distance and costs, and has a high density of public service facilities evenly distributed and perfect, mainly focusing on the daily life of residents. The National Defense Science and Technology University Area is closer to the core area of Dadongmen in terms of positioning and belongs to the residential area. However, unlike the Dadongmen area, the development of station areas in this area is not perfect, and both supply and demand are developing. However, the difference in the “supply-demand” configuration between the two is small. Coupling co-scheduling is better and has certain development potential and prospects.

There are a total of 10 stations belonging to the category of preliminary coordination, accounting for 23% of the total. Their scores on both supply and demand sides are generally below 0.3, indicating a low-level development state. According to the distribution of rail transit station areas (Fig 12c), this type is mainly concentrated in the urban fringe areas, forming a patchy development state with West Qilitang Station, East Qilitang Hefei Railway Station, Xiuning Road Station, and North Wulijing Station as the core. The West Qilitang District and North Wulijing District are influenced by natural geographical environments such as green spaces and rivers, the East Qili Hefei Railway Station District is influenced by planned land and railway tracks, and the Xiuning Road District is mainly industrial land. All three belong to the discontinuous and imperfect development of the road network, reduced residential areas in the station space, incomplete configuration of public service facilities, and inability to carry out diverse station development. They can only focus on meeting the basic living needs of residents, hindering the further development of the station area and causing a moderate imbalance in regional development.

There are a total of 2 stations belonging to the uncoordinated, accounting for 5% of the total. Among them, Xinghua Village Station scored 0.2411, Fangmiao Station scored 0.2234, and the overall scores of the supply and demand sides were around 0.1, indicating a low level of development. According to their distribution (Fig 12d), it can be seen that the severely imbalanced development station areas are all distributed in the urban edge areas. The large green spaces in the Xinghua Village Station area make the road network less networked and reliable, and the road planning system is not mature; On the one hand, Fangmiao Station also has parks and green spaces, but the main reason is that two railway lines are crossing around Fangmiao Station, and corresponding public service facilities cannot be built around the railway. In addition, the nature of the land around Fangmiao Station is mainly industrial and residential land, which further restricts the development of the station area. Under the constraints of these three conditions, Fangmiao Station has become the station with the lowest level of development among various rail transit stations on the Second Ring Road.

### Classification of coupling types and analysis of coupling characteristics

**Fig 13:**
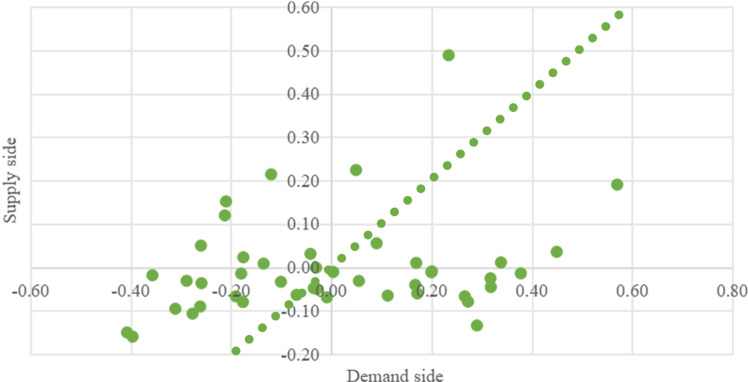
Distribution of the scores of the demand side and the supply side.

By utilizing the relationship between supply side A and demand side B, they are divided into three types: Supply Lag type, Supply-Demand Synchronization type and Demand Lag type.Based on the calculation results, the coupling types of each rail transit station area on the Hefei Second Ring Road are divided (Fig 13). Among them, there are 11 “supply-demand” comprehensive results located above X = Y, and the service supply level of their station areas is greater than the demand level of the community living area space function, belonging to the demand lag type rail transit station area; There are 29 rail transit stations located below X = Y, and their level of functional development is not sufficient to support the demand for space in the community’s living area, belonging to a supply lag type rail transit station area; There are approximately 4 points near the X = Y reference line, indicating that the demand level and service support level of the population in 4 rail transit station areas are relatively balanced, belonging to supply-demand synchronization type of rail transit station area.

**Fig 14:**
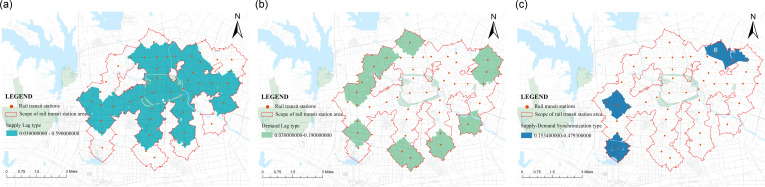
The distribution of stations of each rail transit station area.

According to the spatial distribution of various types of rail transit station areas (Fig 14), the spatial distribution of coupling differences in the comprehensive results is relatively concentrated. The distribution of the difference in comprehensive indicators between the supply and demand sides of the supply lag typerail transit station area is relatively regular, forming a spatial unit with the Sanxiaokou Dadongmen commercial district as the center, north to the Haitang area, and south to the Third Hospital of the city Zhugang area. The supply system of this spatial unit is relatively lagging, and the demand level of the population is much higher than the station service support level, and there is a clear clustering phenomenon. The supply and demand difference of the Sanxiaokou Sipailou commercial district is the largest, indicating that its regional economic development level is high and the population clustering degree is high. However, the construction measures related to the functional organization and development of the community living area have not been followed up, and the configuration of public service facilities cannot meet a large number of people’s needs in the area. There is a large gap between supply and demand. There is a phenomenon of imbalanced development.

The distribution of demand lag type rail transit station areas has no obvious regularity, mainly distributed in the edge areas of urban areas, with obvious agglomeration phenomenon on the northwest side. The demand lag type rail transit station areas with significant differences are mainly concentrated on the south side. Influenced by Hefei South Station, station area development is mainly to meet the basic living needs of the mobile population, not to meet personal and social development as the development direction. Therefore, it has limitations in economic development. In the future, by improving the walking accessibility of the station area, the high-speed rail station can smoothly connect with the surrounding urban areas, quickly handle the pedestrian flow brought by the high-speed rail station, make reasonable use of the pedestrian flow aggregation effect, and revitalize the station and surrounding areas while improving the vitality of the station area.

Although the supply-demand synchronization typerail transit station area is in a state of balanced development between supply and demand, the overall level of development is not high. To become a rail transit station area with high construction completion, good vitality concentration, and matching urban functions with node areas, it is still necessary to make reasonable use of the passenger flow brought by rail transit, revitalize the surrounding economy through appropriate station area development, and promote the mode of crowd gathering and station area development again. Gradually, it is moving towards a positive cycle of “crowd residence station area development economic development”, so that the coupling degree of demand for rail transit station areas and the level of rail transit service support steadily moves towards a more perfect development stage.

## Conclusion

This study systematically analyzes the supply-demand matching situation in Hefei’s rail transit station areas by constructing an evaluation model for the degree of supply-demand coupling coordination. The results reveal disparities in the spatial distribution of supply-demand coordination within these areas. Overall, the supply-demand coordination is higher in the central urban regions, while varying degrees of supply-demand imbalance are evident in the peripheral areas. This finding indicates that the degree of supply-demand matching in urban rail transit station areas is significantly influenced by spatial location, economic vitality, and the availability of public service resources. By identifying the regions with oversupply or undersupply, scientific support is provided for the further planning, configuration, and sustainable development of TOD in the future. Specifically:For those regions where the service level of the rail transit station area fails to meet the needs of the population, that is, in a state where the supply is less than the demand, the service level on the supply side can be improved by enhancing public services and improving transportation infrastructure, so as to elevate the overall service quality and enhance the travel experiences of residents. In this way, a further balance between supply and demand can be achieved. As for the regions where the service level of the rail transit station area exceeds the needs of the population, namely, in a state where the supply is greater than the demand, it is necessary to put the relevant TOD planning on hold. Measures should be taken to boost the economic vitality of these regions and attract more population inflows. By enhancing the demand capacity of the permanent residents on the demand side, the balance between supply and demand can be realized. This research is highly significant for optimizing the planning of urban rail transit station areas and underscores the necessity for tailored approaches in actual planning, emphasizing the allocation of resources in peripheral regions to progressively achieve coordinated development between central and peripheral areas.

By adjusting the mutual alignment and dynamic coupling between human demand and TOD planning configurations, the method aims to fulfill people’s needs for an improved quality of life while achieving optimal overall performance. In the initial stage of TOD planning, the location for development is determined by inferring the service demand of the local population and identifying transportation corridors, thereby establishing the overall layout structure of rail transit. During the intermediate stage, the development status of TOD planning is monitored by assessing the dynamic coupling between the transportation functions and the performance of rail transit station areas under the TOD model. This assessment helps determine the city’s development stage and allows for adjustments based on the implementation status of superior planning policies. In the final stage, TOD planning governance is refined by adjusting the TOD planning model in response to spatially uneven and mismatched phenomena between the supply of rail transit station areas and human demand. Land use and transportation policies are employed to address the conflicts arising from traffic congestion and land scarcity during urban development.

Due to the potential impact of data timeliness and accuracy on model evaluations, as well as the possibility that selected indicators may not encompass all influencing factors during generalization, it is hoped that future research will build upon this study to enhance its universality and practical value. This will provide scientific support for the sustainable development of rail transit systems in large cities.
